# Exploring the Associations between Media and Instagram Interaction Patterns with Weight Bias among Undergraduate Nutrition Students in the Brazilian Nutritionists’ Health Study

**DOI:** 10.3390/nu16142310

**Published:** 2024-07-18

**Authors:** Pabyle Alves Flauzino, Valéria Troncoso Baltar, Leticia Radin Pereira, Shelly Russell-Mayhew, Antonio Augusto Ferreira Carioca

**Affiliations:** 1 Graduate Program in Public Health, Ceará State University, Av Dr Silas Munguba 1700, Fortaleza 60714-903, CE, Brazil; pabyllef@gmail.com; 2 Department of Epidemiology and Biostatistics, Fluminense Federal University, Travessa Marquês de Paraná, 303/3 Floor Center, Niterói 24030-210, RJ, Brazil; vtbaltar@id.uff.br; 3 Department of Community Health Sciences, Cumming School of Medicine, University of Calgary, 3280 Hospital Drive NW, Calgary, AB T2N 4Z6, Canada; leticia.radinpereira@ucalgary.ca; 4 Werklund School of Education, University of Calgary, 2500 University Drive NW, Calgary, AB T2N 1N4, Canada; mkrussel@ucalgary.ca; 5 Graduate Program in Public Health, University of Fortaleza, 1321 Washington Soares Avenue, Fortaleza 60811-905, CE, Brazil

**Keywords:** nutrition students, social media, weight bias, body image, Brazil, SATAQ-3

## Abstract

This study examined the association between media and Instagram interaction patterns with weight bias among undergraduate nutrition students in the Brazilian Nutritionists’ Health Study. We also explored the potential mediating role of students’ own body image perception in these relationships. A total of 406 students (78% women) participated in this cross-sectional analysis. Sociodemographic data, media influence, Instagram interaction patterns, body image perception, and weight bias were assessed using semi-structured questionnaires. Findings indicated that exposure to fitness content on Instagram (*β* = 0.17, *p* < 0.001) and the pursuit of an ideal athletic body (*β* = 0.12, *p* = 0.034) were associated with increased weight bias. In contrast, engagement with body diversity content (*β* = −0.23, *p* < 0.001) and perceived pressure from media to conform to appearance ideals (*β* = −0.24, *p* < 0.001) had a mitigating effect on weight bias. Notably, body image perception did not mediate these relationships (*p* > 0.05). In conclusion, this study revealed a link between media exposure and weight bias among undergraduate nutrition students, independent of their body image perception. Developing social media literacy programs that encourage students to critically evaluate media content is imperative to reduce weight bias. Additionally, a deeper examination of the media content that contributes to weight bias and the potential need for targeted regulatory measures is warranted.

## 1. Introduction

Weight bias refers to negative beliefs, attitudes, assumptions, and judgments in society towards individuals living with obesity [1]. Weight stigma is the expression of this bias, manifested through harmful social stereotypes directed at people living with obesity [1]. Experiencing weight stigma can lead to adverse effects, including compromised psychosocial well-being, heightened risk factors for depression, increased metabolic risks, and reduced self-esteem [2]. For instance, a meta-analysis [3] found an association between greater exposure to weight stigma and worse mental health outcomes in young individuals. Similarly, a prospective study involving 986 adults [4] demonstrated that experiencing weight stigma leads to multisystem physiological dysregulation, doubling the 10-year risk of experiencing high allostatic load—a predictor of morbidity and mortality.

The media often perpetuates weight bias by influencing individuals’ body image perception [5], defined as the accuracy with which one perceives one’s own body size [6]. This is mainly driven by the portrayal of thin and athletic bodies as the ideal [7], alongside the suggestion that body weight is entirely within an individual’s control [8]. Despite the prevalence of content focusing on the thin ideal, particularly on social networks like Instagram, there has been a recent surge in content addressing body diversity [9,10]. Wanniarachchi and colleagues [10] conducted a systematic review to identify key themes regarding weight stigma on social media platforms, highlighting the ongoing tension between the perpetuation of the thin ideal and the rising visibility of body diversity content. These studies collectively underline the nuanced relationship between social media, the perpetuation of weight bias, and the emerging counter-movements advocating for body diversity. Consequently, Instagram’s interaction patterns, including the type of weight-related content accessed (thin ideal vs. diverse body types), the duration of platform use, and its role as a source of weight-related information, may distinctly impact weight bias [11,12], emphasizing the need for further exploration into these complex relationships.

Exposure to media may influence body image in university students [13,14], particularly among nutrition students who may be at higher risk for weight-related issues compared to the general population or non-nutrition students [15,16,17,18]. A systematic review conducted by Rounsefell and colleagues [19] found that social media engagement and exposure to image-related content adversely affect body image and food choices among healthy young adults. Considering that stigma towards others is linked to individual characteristics [20,21], it is plausible that one’s own body image perception might mediate the association between media and weight bias among nutrition students.

In light of the pervasive weight bias observed in the broader context and the potential influence of media on shaping these biases, it becomes imperative to understand the specific dynamics at play, especially among nutrition students. As future professionals who might support clients living with obesity, understanding the associations between sociocultural factors, media patterns, and individual perceptions with weight bias is crucial. Thus, our study employs a structural equation model to assess not only the associations between media and Instagram interaction patterns with weight bias but also to explore the mediating role of body image perception in these relationships among undergraduate nutrition students.

## 2. Materials and Methods

### 2.1. Study Design and Participants

This is a cross-sectional analysis of the Nutritionists’ Health Study (NutriHS) [22], carried out between 2019 and 2020 in the city of Fortaleza, Ceará, Brazil. NutriHS is a multicenter cohort study comprising the University of São Paulo, the State University of Campinas, the University of Fortaleza, and the Ceará State University aiming at monitoring the health status of nutrition students and professionals in Brazil. 

For this study, only students attending a higher education institution in the State of Ceará were included. We used a convenience sampling method and conducted recruitment between June 2019 and March 2020. Recruitment strategies included in-person visits to classrooms and Instagram ads on the study’s official profile (@nutrihsceara). To be eligible for inclusion in the study, participants had to be undergraduate nutrition students actively attending classes at any semester in a higher education institution. Both female and male individuals aged 18 years or older were considered eligible, while pregnant or lactating individuals were excluded from participation. Survey participation was facilitated through a dedicated survey website (http://www.fsp.usp.br/nutrihs, accessed on 2 March 2020), where participants created non-transferable personal login accounts. The minimum sample size of 384 individuals was determined using a calculation based on an infinite population, assuming a 50% prevalence of weight bias (the outcome), with a 95% confidence interval. The study was approved by the Ethics Committee of Ceará State University (95402618.3.0000.5534, 28 September 2018), and informed consent was obtained from all participants involved in the study before data collection.

### 2.2. Sociodemographic and Anthropometric Variables

Sociodemographic variables were self-reported and comprised age (years), sex (female or male), race/skin color (White or Black/Brown), family income (<5 minimum wages or ≥5 minimum wages), semester of enrollment (≤fourth semester (first half of the nutrition course) or ≥fifth semester (second half of the nutrition course)). Anthropometric measurements included self-reported body weight (kg) and height (m), which were used to calculate body mass index (BMI; kg/m^2^).

### 2.3. Explanatory Variables

#### 2.3.1. Internalization of Media Appearance Ideals

The Sociocultural Attitudes Towards Appearance Questionnaire–3 (SATAQ-3) was used to evaluate the internalization of media appearance ideals [23]. This instrument, validated for the Brazilian population [24], includes 30 items presented on a five-point Likert scale, ranging from 1 (“completely disagree”) to 5 (“completely agree”). The questionnaire is structured into four distinct subscales: (1) Internalization-General: Assess the extent to which individuals internalize an ideal body (nine items), (2) Internalization-Athlete: Measures the degree of internalization of an athletic body (five items), (3) Pressures: Examines perceived societal pressures arising from the exposure to media to conform to appearance ideals (seven items), and (4) Information: Explores the role of media as an informational source concerning appearance ideals (nine items). A composite score, indicative of the overall internalization of media appearance ideals, was derived by summing the responses from each subscale. Higher scores represent a more pronounced internalization of media appearance ideals. In this study, the Cronbach’s α for SATAQ-3 was 0.91.

#### 2.3.2. Instagram Interaction Patterns

The assessment of Instagram interaction patterns involved the measurement of different variables, including usage time, number of followers, frequency of exposure to content related to fitness and body diversity, and the utilization of Instagram as a source of weight-related information. Participants self-reported the time spent on Instagram over the previous week, utilizing the data retrieved from the Instagram app (Instagram app > “your activity” menu > “daily average use of the last week”). Weekly hours were subsequently converted into daily hours. The number of followers was directly recorded from the Instagram app (Instagram app > number of followers). A structured questionnaire utilizing a five-point Likert scale with responses ranging from 1 (“Never”) to 5 (“Always”) was administered to quantify the frequency of exposure to content related to fitness and body diversity. For fitness content: (1) “I receive fitness-related content (e.g., low-calorie diets, thin or athletic bodies, exercises for hypertrophy or weight loss)”, and (2) “I receive content implying that thinness is synonymous with being healthy”. For body diversity content: (1) “I receive content showcasing body diversity (e.g., diverse bodies sizes, health at every size, weight neutrality, or body-positive movement)”, and (2) “I receive content emphasizing that body weight does not always predict health”. Additionally, participants were queried about the frequency with which Instagram content served as a source of information about what constitutes a healthy body. Responses were provided using the same Likert scale previously described.

### 2.4. Mediating Variable

#### Body Image Perception

Body image perception, defined as how accurately one identifies their body image in relation to their actual body weight [6], was assessed using the Brazilian Silhouette Scale [25]. This figure rating scale, designed and validated for the adult Brazilian population [25,26], contains 15 silhouette figures for each sex arranged in ascending order, each corresponding to a specific mean BMI ranging from 12.5 to 47.5 kg/m^2^. Previous studies have used this scale to measure body image perception among the Brazilian population [27,28,29]. Briefly, after silhouette figures were presented, participants were instructed to indicate which silhouette most closely represented their body size on that day (perceived silhouette). The corresponding number of the silhouette (ranging from 1 to 15) chosen by individuals was recorded. The actual silhouette was determined by converting the actual BMI value (self-reported assessment) into the corresponding silhouette figure. Body image perception was assessed by the difference between the perceived silhouette and the actual silhouette (body image perception = perceived silhouette minus actual silhouette). A resultant value of zero indicated “No distortion”, a value less than zero indicated “Underestimation of body size”, and a value greater than zero indicated “Overestimation of body size”. In this study, the Cronbach’s α for the Brazilian Silhouette Scale was 0.93.

### 2.5. Response Variable

#### Weight Bias

Negative attitudes toward body weight were assessed using the Antifat Attitudes Test (AFAT), a tool that has been translated and validated for the Brazilian population [30]. The scale comprises 34 questions and is structured in a five-point Likert format, with responses ranging from 1 (“Strongly disagree”) to 5 (“Strongly agree”). Weight bias was assessed through three dimensions: (1) Social/Character Disparagement: Encompassing items that describe socially undesirable personality traits and societal disregard for individuals living with obesity (15 items), (2) Physical/Romantic Unattractiveness: Reflecting the perception that individuals living with obesity are deemed unattractive and unacceptable as romantic partners (10 items), and (3) Weight Control/Blame: Representing beliefs pertaining to self-responsibility and self-control in managing body weight (9 items). The scoring mechanism is indicative of the severity of weight bias, with higher scores representing a more pronounced weight bias [30]. In this study, the Cronbach’s α for AFAT was 0.85.

### 2.6. Data Analysis

The Kolmogorov–Smirnov test and graphical analysis (histogram) were employed to assess the normal distribution of continuous variables. Descriptive statistics for continuous variables were presented as median and 25th to 75th percentile, while categorical variables were expressed as absolute and relative frequencies (%). Participants’ characteristics associated with the outcomes (weight bias, AFAT scores) were further stratified by tertiles for a detailed sample description, with tertile 1 representing participants with less weight bias. Comparisons of indicators between AFAT scores tertiles were conducted using appropriated statistical tests: the Mann–Whitney U and Kruskal–Wallis for continuous variables, and the chi-square test for categorical variables. Statistical significance was defined by a *p* value of <0.05. R software, the Lavaan package (version 4.3.2; R Core Team, Vienna, Austria) was used.

#### 2.6.1. Latent Variables: Instagram Interaction Patterns

Factor analysis using the main component was employed to derive the Instagram interaction patterns. Sampling adequacy was assessed using Bartlett’s sphericity test and the Kaiser–Meyer–Olkin (KMO) index, with KMO values exceeding 0.50 and *p* < 0.05 considered acceptable. The following steps were taken to determine the number of factors (standards) retained: (1) applying the initial criteria for eigenvalues > 1.0, and (2) conducting a scree plot analysis followed by an interpretation of each factor. The varimax rotation method was utilized to enhance the interpretability of these factors. Instagram interaction patterns scored in each factor were based on Instagram usage time, number of followers, frequency of exposure to content related to fitness and body diversity, and the utilization of Instagram as a source of weight-related information (factorial load ≥ 0.6 or ≤−0.6). Subsequently, standardized factor score coefficients were estimated using regression analysis.

The factor analysis yielded three interaction patterns to characterize Instagram interaction patterns. Interaction Pattern 1—Fitness Pattern: Associated with the frequency of exposure to “Fitness-related content” (0.816) and “Thinness is synonymous with being healthy” (0.825). Interaction Pattern 2—Body Diversity Pattern: Associated with the frequency of exposure to “Body diversity-related content” (0.834), “Body weight does not always predict health” (0.857), and “Instagram content as a source of information about what constitutes a healthy body” (0.662). Interaction Pattern 3—High Engagement Pattern: Associated with “Instagram daily usage time” (0.810), and the “Number of followers on Instagram” (0.671). Further information on factor analysis and other explanatory variables associated with the patterns can be found in previous publications [31].

#### 2.6.2. Structural Equation Model

The structural equation model (SEM) estimates the strength of all hypothesized relationships between variables within a theoretical framework, encompassing both direct and indirect pathways [32]. To capture the multidimensional nature of the sociocultural questionnaire, we only included Subscale 2 (Internalization-Athlete) and Subscale 3 (Pressures) in the SEM. Within this analysis, the SEM accounted for the following: (1) Latent variables: Instagram interaction patterns (i.e., Fitness Pattern, Body Diversity Pattern, and High Engagement Pattern), (2) Explanatory variables: Societal influence of media on appearance ideals (i.e., Internalization-Athlete, and Pressures), (3) Mediating variable: Body image perception (a continuous variable), and (4) Response variable: Weight bias. When body image perception was used as the “response variable”, its interpretation was treated as ordinal (No distortion > underestimation > overestimation). All continuous variables were standardized, with the standardized coefficients expressing the effect of the exposure on the outcome variable in standard deviation units.

The root-mean-square error of approximation was employed to evaluate the model’s goodness of fit, with an upper limit of the 90% confidence interval < 0.08 indicative of good model quality. Additionally, Comparative Fit Index and Tucker–Lewis Index values > 0.90 were considered indicative of a good-fitting model. Model modification index tests were used to identify significant correlations between the residuals and the variables that could enhance the model performance. The conceptually derived model was tested using the R software, the Lavaan package.

## 3. Results

Out of the 28 nutrition higher education institutions in the State of Ceará, 11 showed interest in participating in the study, resulting in a total of 406 participants. Study participants were predominantly female (78%), enrolled in the fifth semester or beyond (58.3%), with a median BMI of 23.4 (21.2–26.1) kg/m^2^. The association between sociodemographic and academic characteristics by weight bias tertiles are detailed in Table 1. Weight bias was associated with age (*p* = 0.029) and sex (*p* = 0.014).

Table 2 outlines the association between the internalization of media appearance ideals, Instagram interaction patterns, and body image perception by weight bias tertiles. Internalization-General was higher in tertile 1 compared to tertile 2 (*p* = 0.045). Perceived pressure from media to conform to appearance ideals was higher in tertile 1 than tertiles 2 and 3 (both *p* < 0.001). The Body Diversity Pattern exhibited lower scores across the weight-bias tertiles (*p* < 0.001), while the Fitness Pattern showed higher scores (*p* < 0.001).

Table 3 includes data on the relationships between the internalization of media appearance ideals, Instagram interaction patterns, and weight bias, by body image perception categories. Individuals who overestimated their body image exhibited higher perceived pressure from media to conform to appearance ideals compared to those who underestimated it (*p* = 0.001). The fitness interaction pattern was found to be higher in individuals who underestimate their body image compared to those who overestimate it (*p* = 0.004).

**Figure 1:**
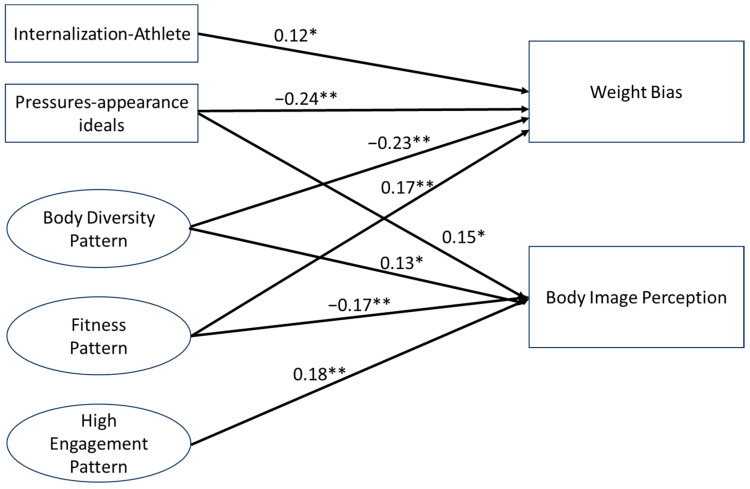
Structural equation model using the internalization of media appearance ideals (i.e., Internalization-Athlete and Pressures) and Instagram interaction patterns (i.e., Body Diversity, Fitness, and High Engagement) as latent variable and predictors of weight bias, and body image perception as mediating variable. Note: The model was adjusted for sex and age. Results are presented as standard estimates. * *p* < 0.05; ** *p* < 0.001. Only statistically significant associations are displayed in the figure. The goodness-of-fit indices indicated an acceptable fit of the SEM (Comparative Fit Index of 1.00, Tucker–Lewis Index of 0.83 and root-mean-square error of approximation, RMSEA of zero (CI 90%: 0, 0.078)).

The SEM results are depicted in Figure 1, with variables obtained from questionnaires presented in rectangles, and variables estimated through factor analysis represented by ellipses. Both the internalization of media appearance ideals and Instagram interaction patterns were found to be associated with weight bias. In detail, Internalization-Athlete exhibited a positive association with weight bias (*β* = 0.12, *p* = 0.034), while Pressures demonstrated an inverse association (*β* = −0.24, *p* < 0.001). The Body Diversity Pattern was inversely associated with weight bias (*β* = −0.23, *p* < 0.001), while the Fitness Pattern had a positive association (*β* = 0.17, *p* < 0.001). Body image perception was positively associated with Body Diversity (*β* = 0.13, *p* = 0.024), and High Engagement Patterns (*β* = 0.18, *p* = 0.008), and negatively associated with Fitness (*β* = −0.17, *p* < 0.001). Body image perception did not mediate the relationship between the internalization of media appearance ideals and Instagram interaction patterns with weight bias (*p* > 0.05).

## 4. Discussion

Our findings revealed a direct association between exposure to fitness content on Instagram and internalization of the ideal athletic body with weight bias, while engagement with body diversity content and perceived pressure from media to conform to appearance ideals had an inverse association with weight bias. Interestingly, even though body image perception was associated with specific Instagram interactions patterns, it did not mediate the association between media and Instagram interaction patterns with weight bias.

The direct association between exposure to fitness content and weight bias aligns with the prevalence of such content in both traditional media and social networks, emphasizing body transformations, often portraying thin-athletic body as ideal [33]. This content tends to oversimplify the complex nature of weight control, attributing it solely to individual willpower and overlooking other determinants such as environmental and genetic factors [5]. Indeed, the belief that body weight management is a straightforward task is linked to increased negative attitudes and biases related to weight across diverse populations [34,35,36]. This association stands as a plausible hypothesis for the observed relationships in our study. Similarly, the internalization of the ideal athletic body had a direct association with weight bias, highlighting the potential influence of media in perpetuating the fear of body fat and promoting the thin-athletic body as the desirable norm [37]. Vartanian and colleagues [38] examined negative attitudes and beliefs toward fatness in individuals adhering to a restrictive diet, revealing that those internalizing the thin-body exhibited stronger negative anti-fat attitudes and beliefs. While an awareness of the causes and consequences of obesity has been shown to mitigate weight bias [39], this phenomenon does not seem prevalent among health students. This highlights the need to integrate learning opportunities on weight bias into the curriculum for nutrition students.

Conversely, engagement with body diversity content exhibited an inverse association with weight bias. Originating as a counter-hegemonic response on social networks, the body diversity movement challenges prevailing norms, promoting diversity in body representation [11]. Our hypothesis, rooted in the “Contact Hypothesis”, suggests that exposure to non-stereotyped and humanized portrayals of diverse bodies fosters empathy, respect, and knowledge, thereby reducing weight bias [40,41,42]. Additionally, the Health at Every Size^®^ movement, embedded in the body diversity content, advocates for a shift away from weight-centric approaches, potentially contributing to weight bias reduction [7,43]. Perceived pressure from media to conform to appearance ideals demonstrated an inverse association with weight bias, indicating that individual experiences with societal pressure about appearance may shape attitudes towards diverse body sizes. This observation aligns with and can be elucidated by the social identity theory [44]. Students experiencing pressure to conform to societal expectations of a thin-ideal body may identify with socially oppressed groups, potentially reducing their likelihood of holding negative attitudes toward individuals with different body types, especially if they do not possess a thin-ideal body themselves.

Contrary to our initial hypothesis, body image perception did not mediate the relationship between the internalization of media appearance ideals, Instagram interaction patterns, and weight bias. This finding implies that exposure to and internalization of media ideals might be directly associated with attitudes toward weight, potentially by reinforcing societal weight norms or stereotypes, without necessarily altering individuals’ perceptions of their own bodies. While body image perception is associated with media and interactions on platforms like Instagram, its role in mediating the translation of these associations to weight bias appears to be less direct than previously hypothesized. The construct of body image is captured by two main dimensions: attitudinal and perceptual [6,45]. The attitudinal dimension involves the satisfaction one has with the size or shape of one’s body and the view of the body as being pleasing or displeasing (i.e., body image satisfaction or body image dissatisfaction). This dimension includes three main components: affective, cognitive, and behavioral, each measuring different aspects of body image [46]. The perceptual dimension of body image (i.e., body image perception) refers to the awareness or knowledge of the size of the body and the accuracy with which one perceives one’s body size, and it can be classified by the presence or absence of distortion in identifying body size [6,45]. Our study only measured the perceptual dimension of body image, and the lack of mediating effects on body image perception found in our study may suggest the involvement of other psychological variables or mediating factors such as self-esteem, mental health status, or more nuanced aspects of body image dimensions that our study may not have fully captured. Future research could benefit from employing multidimensional measures of body image that not only assess body image perception but also the attitudinal dimensions of body image, including the affective, cognitive, and behavioral components [46], to provide a more comprehensive understanding of the complex pathways through which media exposure translates to weight bias. Such insights could be crucial in developing more effective health promotion and media literacy programs that address these direct associations.

Our findings highlight the importance of recognizing that undergraduate nutrition students, throughout their academic journey, may benefit from guidance on navigating content related to weight bias and health, particularly on social media platforms like Instagram. Integrating tailored social media literacy programs into nutrition education might effectively help future nutrition professionals critically evaluate content and address weight bias. Emphasizing the importance of diverse body representations and promoting healthy attitudes towards weight could serve as central themes in these programs [47]. Moreover, developing skills in navigating social media discussions about body image and obesity may empower future professionals to approach weight-related issues with sensitivity, informed perspectives, and the ability to provide empathetic and unbiased care.

Additionally, our research highlights the significant association between media and weight bias, emphasizing the need for targeted regulatory measures. These findings indicate that the type of content consumed is associated with societal attitudes towards body weight. Therefore, it is crucial to advocate for policies that regulate media content, especially concerning how body images and ideals are presented. Some countries already have regulations regarding media content, especially concerning advertising. For instance, France requires magazines to label photoshopped images if they alter the appearance of models [48], and the UK has rules against misleading or socially irresponsible body image advertisements [49]. Implementing these regulations might mitigate the adverse effects of media on public perceptions of body weight, promoting a healthier and more inclusive social environment.

This study has several strengths. First, the utilization of SEM enhances the robustness of our data analysis, offering a more comprehensive approach than conventional techniques. SEM facilitated the theoretical testing of multiple, simultaneous relationships among explanatory, mediating, and response variables. Second, the innovative creation of Instagram interaction patterns from latent variables adds depth to our understanding of subjects’ interactions within the social network. This approach sensitizes the analysis, capturing the nuanced dynamics of Instagram usage patterns. Third, this study pioneers the evaluation of a new form of social media, Instagram, in relation to weight bias. Studying Instagram interaction patterns helps us understand how the platform is associated with attitudes toward weight.

It is essential to acknowledge certain limitations in our study. The measurement of weight bias may be prone to response biases, such as conscious perceptions of shame or guilt, which could influence participant responses. Nevertheless, the questionnaire is validated and exhibited robust psychometric indices [30]. Another limitation is related to the lack of formal validation for the questionnaire administered to assess students’ interaction patterns on Instagram. Future studies may benefit from a validated instrument for a more rigorous assessment of Instagram usage patterns. Participants self-reported their body weight and height, which may limit their ability to accurately estimate their body size and affect the validity of the mediating variable (i.e., body image perception calculated from self-reported BMI). Despite this limitation, previous studies [50,51] suggest that self-reported weight and height are reliable for calculating BMI in adults, providing a reasonable basis for our measurements. Given that this analysis is cross-sectional in nature, it is important to note that causal inferences cannot be made. Lastly, caution is warranted in generalizing our results beyond our sample, as it is relatively small and primarily comprises young female undergraduate nutrition students. Future research should aim for diverse participant populations to enhance the external validity of findings.

## 5. Conclusions

In conclusion, our study highlights the associations between media and Instagram interaction patterns with weight bias, independent of body image perception. The pursuit of the ideal athletic body and regular exposure to fitness content on Instagram directly contribute to increased weight bias. Conversely, perceived pressure from media to conform to appearance ideals and active engagement with body diversity content mitigate such bias. Future research could benefit from exploring additional variables that might mediate these relationships, or from employing longitudinal designs to better understand the directionality and causality of these associations. To counteract weight bias among undergraduate nutrition students, social media literacy initiatives encouraging students to critically evaluate content on platforms like Instagram are crucial. Additionally, a deeper examination of the specific media content contributing to weight bias is warranted. This may highlight the potential need for targeted regulatory measures, which could mitigate the adverse effects of media on public perceptions of body weight and promote a healthier, more inclusive social environment.

## Figures and Tables

**Table 1:** Sociodemographic and academic characteristics by weight bias tertiles in undergraduate nutrition students (*n* = 406).

	Weight Bias			*p*
	Tertile 1 (*n* = 143)	Tertile 2 (*n* = 123)	Tertile 3 (*n* = 140)	
Age (years)	22 (20–24)	23 (21–27) ^1^	23 (20–27)	0.029
Female	123 (86.0)	93 (75.6)	101 (72.1) ^1^	0.014
Body mass index (kg/m^2^)	23.4 (21.0–25.8)	23.4 (21.2–26.1)	23.7 (21.3–26.6)	0.608
Skin color				
White	70 (48.9)	58 (47.2)	64 (45.7)	0.954
Black/Brown	69 (48.3)	61 (49.6)	70 (50.0)	
Do not know	4 (2.8)	4 (3.3)	6 (4.3)	
Family income (Brazilian minimum wage) *				0.896
<5 minimum wages	99 (69.2)	82 (66.7)	94 (67.1)	
≥5 minimum wages	35 (24.5)	32 (26.0)	33 (23.6)	
Do not know	9 (6.3)	9 (7.3)	13 (9.3)	
Semester of enrollment				
≤fourth semester	58 (40.6)	49 (39.8)	62 (44.3)	0.727
≥fifth semester	85 (59.4)	74 (60.2)	78 (55.7)	

Results are presented as median (25th–75th percentile) or n (%). Tertile 1 indicates less weight bias. ^1^ Indicates results significantly differed from Tertile 1 (*p* < 0.05). * Brazilian minimum wage (equivalent to USD 253 per month).

**Table 2:** Internalization of media appearance ideals, Instagram interaction patterns, and body image perception by weight bias tertiles in undergraduate nutrition students (*n* = 406).

	Weight Bias			*p*
	Tertile 1 (*n* = 143)	Tertile 2 (*n* = 123)	Tertile 3 (*n* = 140)	
Internalization of media appearance ideals	103.8 (80.9–116.1)	90.0 (69.3–113.4) ^1^	97.0 (77.7–110.5)	0.028
Internalization-General	27 (17–33)	22 (16–31) ^1^	26 (19–31)	0.045
Internalization-Athlete	15 (11–19)	14 (10–19)	15 (12–19)	0.456
Pressures	27 (19–31)	21 (13–28) ^1^	21 (14–25) ^1^	<0.001
Information	23 (18–30)	23 (17–30)	26 (21–30)	0.097
Instagram interaction patterns				
Body diversity	0.49 (−0.26–1.13)	0.12 (−0.40–0.70) ^1^	−0,32 (−0.97–0.29) ^1,2^	<0.001
Fitness	−0.20 (−0.93–0.38)	−0.00 (−0.62–0.68)	0.18 (−0.45–0.90) ^1^	<0.001
High engagement	−0.12 (−0.67–0.38)	−0.29 (−0.81–0.41)	−0.25 (−0.77–0.45)	0.508
Body image perception				0.116
No distortion	12 (8.4)	15 (12.2)	21 (15.0)	
Underestimation	30 (21.0)	34 (27.6)	41 (29.3)	
Overestimation	101 (70.6)	74 (60.2)	78 (55.7)	

Results are presented as median (25th–75th percentile) or *n* (%). Tertile 1 indicates less weight bias. ^1^ Indicates results significantly differed from Tertile 1 (*p* < 0.05). ^2^ Indicates results significantly differed from Tertile 2 (*p* < 0.05).

**Table 3:** Internalization of media appearance ideals, Instagram interaction patterns, and weight bias by body image perception categories in undergraduate nutrition students (*n* = 406).

	Body Image Perception			*p*
	No Distortion (*n* = 48)	Underestimation (*n* = 105)	Overestimation (*n* = 253)	
Internalization of media appearance ideals	100.02 (76.3–113.5)	93.4 (73.7–111.1)	98.6 (76.5–115.1)	0.384
Internalization-General	27 (20–32)	23 (16.5–31.5)	26 (17.5–32)	0.444
Internalization-Athlete	15 (12–18)	16 (11–20)	14 (10–18)	0.133
Pressures	21 (17–26.7)	20 (13–26)	24 (17–29) ^2^	0.001
Information	25.5 (19–30.7)	25 (18.5–29)	24 (19–30)	0.947
Instagram interaction patterns				
Body diversity	−0.22 (−1.16–0.58)	−0.02 (−0.62–0.59)	0.12 (−0.43–0.82) ^1^	0.025
Fitness	0.18 (−0.73–1.12)	0.20 (−0.42–0.74)	−0.13 (−0.82–0.49) ^2^	0.004
High engagement	−0.25 (−0.85–0.30)	−0.28 (−0.74–0.31)	−0.18 (−0.72–0.76)	0.122
Weight bias	50 (45.25–60)	51 (44–58)	47 (43–55.5)	0.059
Social/Character disparagement	17 (16–21)	17 (16–19.5)	16 (15–19)	0.069
Physical/Romantic unattractiveness	15 (13–20)	16 (14–19.5)	15 (13–18)	0.256
Weight control/blame	18 (15–21)	17 (13–21)	15 (13–20)	0.050

Results are presented as median (25th–75th percentile). Tertile 1 indicates less weight bias. ^1^ Indicates results significantly differed from Tertile 1 (*p* < 0.05). ^2^ Indicates results significantly differed from Tertile 2 (*p* < 0.05).

## Data Availability

The data presented in this study are available on request from the corresponding author due to privacy, ethical, and legal reasons.
